# Benign Paroxysmal Positional Vertigo After Joint Replacement Surgeries: Case Series

**DOI:** 10.7759/cureus.51839

**Published:** 2024-01-08

**Authors:** Khalid Bashir, Abdulla Yousuf, Talha Shahzad, Keebat Khan, Zeenat Khuda Bakhsh

**Affiliations:** 1 Emergency Medicine, Hamad General Hospital, Doha, QAT; 2 Emergency Medicine, College of Medicine Qatar University, Doha, QAT; 3 Medical Education and Simulation, Hamad General Hospital, Doha, QAT; 4 Emergency Medicine, University of Central Lancashire, Lancashire, GBR; 5 Emergency Medicine, Hamad Medical Corporation, Doha, QAT

**Keywords:** epley maneuver, dix-hallpike test, vertigo, postoperative complications, anesthesia, knee replacement, hip replacement, joint replacement, bppv

## Abstract

Benign paroxysmal positional vertigo (BPPV) is characterized by brief episodes of vertigo triggered by changes in head position caused by the displacement of otoliths from the utricle to the semicircular canals, particularly the posterior canal. This study explored the potential link between BPPV, the patient's preexisting conditions, and surgery-related factors including surgical positioning, duration of the procedure, exposure to vibratory forces, and anesthesia effects. This report presents two cases of BPPV following major joint replacement surgery. The first case involved a 65-year-old male with a history of diet-controlled diabetes who had undergone right-sided total hip replacement. The second case was that of a 60-year-old female with a history of osteoporosis managed with bisphosphonate therapy and left-sided knee replacement. Both patients developed vertigo symptoms one day postoperatively and were diagnosed with BPPV. In both cases, the Dix-Hallpike test confirmed the right-sided posterior canal BPPV diagnosis, and the patients were successfully treated using the Epley maneuver. Notably, there was no recurrence of vertigo at the four-week follow-up. These cases highlight the importance of considering BPPV in patients presenting with vertigo symptoms after joint replacement surgery, especially in the presence of comorbidities like diabetes and osteoporosis which possibly increase susceptibility to BPPV. This article presents two cases of benign paroxysmal positional vertigo (BPPV) following non-otologic surgery. It explores the pathophysiological mechanism underlying BPPV after such surgeries and also discusses the diagnosis and treatment approaches. This underscores the need for prompt diagnosis and treatment of BPPV to improve postoperative outcomes.

## Introduction

Benign paroxysmal positional vertigo (BPPV) manifests as episodic, intense vertigo episodes, commonly triggered by specific head movements [1]. This condition, primarily affecting the vestibular system, involves the dislodgement of otoliths-minute calcium carbonate crystals from the utricle to the semicircular canals, particularly impacting the posterior canal [2]. The migration of these crystals leads to irregular stimulation of sensory hair cells within the canals during head movements, producing inaccurate neural signals and the sensation of vertigo, a defining feature of BPPV.

To fully comprehend the pathophysiology of BPPV, it's essential to consider some procedure-related factors. These include the patient's head positioning during surgical interventions, the duration of the operation, and exposure to repetitive vibratory and percussive forces [3]. Such factors could prompt the displacement of otoliths into the posterior semicircular canal, triggering an atypical vestibular response, especially notable when the patient assumes an upright position.

The likelihood of developing post-surgical BPPV is also influenced by various patient-related factors, such as dyslipidemia, endocrine and vascular disorders, female gender, and being within the perimenopausal age group (50-60 years) [4]. Additional contributing factors include a history of migraines, previous cranial trauma, autoimmune and neurological disorders, hypovitaminosis, and inner ear inflammation. Understanding these risk factors is crucial for preventing and managing post-surgical BPPV.

Diagnosis of BPPV is primarily established through the Dix-Hallpike test. This diagnostic maneuver involves positioning the patient's head horizontally, with the head overhanging the bed. A positive test, indicated by symptom recurrence and nystagmus, confirms posterior canal BPPV [1]. The most common treatment is the Epley maneuver, a bedside technique designed to remove the dislodged crystals from the semicircular canals. Generally, no further investigations are necessary for BPPV management. However, without timely and appropriate treatment, patients may experience prolonged discomfort and impaired quality of life.

We document two instances of benign paroxysmal positional vertigo (BPPV) in patients following major joint replacement surgeries. The first case involves a 65-year-old male with a medical background of diet-controlled diabetes mellitus, who underwent a right-sided total hip replacement. The second case features a 60-year-old female with a history of osteoporosis, managed with bisphosphonates, undergoing a left-sided knee replacement. Both patients experienced vertigo episodes shortly after their respective surgeries, which were effectively managed in a clinical setting.

## Case presentation

Case 1

Case 1 delineated the clinical journey of a 65-year-old male who underwent right-sided total hip replacement. This patient's medical background, notably a diagnosis of diet-controlled diabetes mellitus, was significant in the context of his postoperative convalescence. The surgical procedure and immediate postoperative period were characterized by the absence of complications. However, on the second postoperative day, while attempting to rise from bed, the patient experienced abrupt and distressing bouts of vertigo, a symptomatology consistent with vestibular dysfunction. These vertiginous episodes were marked by changes in head positioning.

The on-call Ear, Nose, and Throat (ENT) specialist swiftly conducted the Dix-Hallpike test, a diagnostic technique primarily utilized to detect BPPV, especially targeting the posterior semicircular canal. The manifestation of positive nystagmus during this test, a salient symptom indicative of BPPV, led to a diagnosis of posterior canal BPPV. The patient received treatment through the Epley maneuver, a canalith repositioning method aimed at relocating the dislodged otoconia within the inner ear, thereby mitigating vertigo symptoms.

The patient's response to the treatment proved to be efficacious, as evidenced by the cessation of vertigo episodes, thus validating the utility of the Epley maneuver in managing posterior canal BPPV. Following a five-day hospitalization, wherein he received assistance from physiotherapists for mobilization, he was discharged home. Notably, at the three-week follow-up, the patient reported complete resolution of vertigo symptoms, indicative of the absence of recurrent BPPV. This outcome bears particular significance in light of his pre-existing diabetes, which could potentially complicate both the postoperative trajectory and management of vertigo.

Case 2

Case 2 involved a 60-year-old female patient who underwent left-sided knee replacement, a prevalent procedure intended to alleviate pain and restore functionality in knees afflicted by osteoarthritis. The patient's medical history was notable for osteoporosis, for which she was receiving bisphosphonate therapy, a treatment aimed at augmenting bone density and diminishing fracture risk.

After the knee replacement surgery, the patient began experiencing vertiginous symptoms on the first postoperative day, specifically when engaging in head movements, particularly while turning her head to the right. This symptom is characteristic of vestibular disorders. The ENT team's assessment included conducting a Dix-Hallpike test, which confirmed the diagnosis of right-sided posterior canal BPPV through observation of vertigo and torsional nystagmus when the patient's head was tilted to the right.

Following this diagnosis, the patient underwent treatment with the Epley maneuver, a series of targeted head and body movements designed to reposition the dislodged otoconia from the semicircular canal back to the utricle, thereby nullifying vertiginous symptoms. The patient's response to this intervention was positive, as manifested by the relief from vertigo. The patient was subsequently discharged on the sixth postoperative day.

At the four-week follow-up, the patient reported a complete absence of vertigo symptoms, thereby confirming the effectiveness of the treatment (Table 1). Considering her medical history, factors such as osteoporosis, the nature of knee surgery, and the effects of anesthesia might have contributed to the onset of BPPV. This case emphasizes the necessity of a comprehensive approach to evaluate the potential risk factors in postoperative patients, particularly those with intricate medical histories.

**Table 1:** This table concisely presents each case's essential information, including patient demographics, medical history, surgery, symptom onset, diagnosis, treatment, and outcomes.

Case	Age	Medical History	Type of Surgery	Onset of Symptoms	Diagnosis	Treatment	Follow-Up
Case 1	65-year-old male	Diet-controlled diabetes	Right-sided total hip replacement	Second-day post-surgery	Right-sided posterior canal BPPV	Epley maneuver	No vertigo at 3 weeks
Case 2	60-year-old female	Osteoporosis, undergoing bisphosphonate therapy	Left-sided knee replacement	First-day post-surgery	Right-sided posterior canal BPPV	Epley maneuver	No vertigo at 4 weeks

## Discussion

This case article delineates two distinct cases wherein patients developed BPPV after significant joint replacement surgeries. The first case involved a 65-year-old male patient with a medical history of diet-controlled diabetes mellitus who underwent a right-sided total hip replacement. The second case concerned a 60-year-old female patient, with a history of osteoporosis under bisphosphonate treatment, who underwent a left-sided knee replacement.

Joint replacement surgeries are routinely conducted to alleviate pain and enhance functionality in severely arthritic or damaged joints [5,6]. Despite their widespread success, these surgical interventions can potentially lead to an array of complications.

The manifestation of BPPV in the post-operative phase in these cases draws attention to potential associations between the effects of anesthesia, surgical manipulations, and the onset of BPPV [7]. General anesthesia, an integral aspect of these surgical procedures, may exert an influence on the functionality of the inner ear, possibly precipitating the dislodgement of otoliths due to fluctuations in blood flow or pressure within the ear [8].

The prompt diagnosis and subsequent management of BPPV in these patients were instrumental in the amelioration of their symptoms [1]. Utilization of the Dix-Hallpike test for diagnostic purposes affirmed the presence of posterior canal BPPV. Intervention with the Epley maneuver, a therapeutic technique designed to reposition the dislodged otoliths within the inner ear, effectively resolved the vertiginous symptoms [9].

These case studies accentuate the critical importance of early detection and intervention in the treatment of BPPV, particularly in the context of postoperative care [10]. Early therapeutic intervention not only provides symptomatic relief but also substantially diminishes the risk of associated morbidities.

## Conclusions

In conclusion, these cases highlight BPPV as a potential complication following joint replacement surgeries, possibly linked to factors like anesthesia or surgical manipulation. The presence of comorbid conditions like diet-controlled diabetes and osteoporosis in these patients adds complexity to their postoperative course and necessitates a comprehensive approach in managing such cases. The effectiveness of prompt diagnosis and treatment, as seen in these patients, reinforces the need for heightened awareness and timely management of BPPV in postoperative patients, especially those with existing risk factors. Further research is essential to elucidate the precise mechanisms connecting surgical procedures, anesthesia, and the onset of BPPV, thereby enhancing our understanding and management of this condition.
